# Multi-disciplinary treatment of broncho-esophageal fistula in a high-risk single-lung patient

**DOI:** 10.1186/s13019-024-03287-5

**Published:** 2025-01-11

**Authors:** Rune Haaverstad, Kjell Ovrebo, Lorentz Sandvik, Håvard Seland, Gunnar Reksten Husebø, Vegard Skalstad Ellensen, Marit Farstad, Eivind Strandenes, Rajinder Sharma, Marianne Øksnes, Anders Kjellevold Storesund, Solveig Moss Kolseth

**Affiliations:** 1 Section of Cardiothoracic Surgery, Department of Heart Disease, Haukeland University Hospital, Jonas Lies vei 65, 5021 Bergen, Norway; 2 Department of Clinical Science, Faculty of Medicine, University of Bergen, Bergen, Norway; 3 Department of Surgery, Haukeland University Hospital, Bergen, Norway; 4 Department of Otolaryngology, Head and Neck Surgery, Haukeland University Hospital, Bergen, Norway; 5 Department of Plastic and Reconstructive Surgery, Haukeland University Hospital, Bergen, Norway; 6 Department of Thoracic Medicine, Haukeland University Hospital, Bergen, Norway; 7 Department of Anaesthesia and Intensive Care, Haukeland University Hospital, Bergen, Norway; 8 Department of Medicine, Haukeland University Hospital, Bergen, Norway

**Keywords:** Bronchial stent, Broncho-esophageal fistula, Bronchoplasty, Esophagectomy, Esophageal stent, Non-small lung cancer, Thoracoplasty, Tracheobronchial stent, Veno-venous extracorporeal membrane oxygenation

## Abstract

**Background:**

A broncho-esophageal fistula (BEF) is a medical and surgical disaster. Treatment of BEF is often limited to palliative stent treatment that may migrate or cause erosions and tissue necrosis. Surgical repair of BEF is the only established definite treatment.

**Case presentation:**

BEF presented in a 40-year-old female patient 8 years after curative treatment with pneumonectomy and radio-chemotherapy for advanced lung cancer. She had autoimmune comorbidity, a single lung, vocal cord paralysis and an extremely hostile thorax. Multi-disciplinary collaboration, close patient involvement and evaluation by the hospital medical ethics committee were key elements in the following treatment course. After temporary stent treatment, a carefully staged surgical marathon was performed: Veno-venous ECMO was established to secure oxygenation, and bilateral thoracotomy and laparotomy performed to access structures in the frozen mediastinum. After extensive thoracoplasty and high-risk dissection, esophagectomy was performed and the 20 × 35 mm bronchial defect repaired by bronchoplasty with a latissimus muscle flap. It was complicated by thrombotic occlusion of the upper venous system, repeated postoperative bleedings and critical illness neuropathy. The patient recovered and was discharged 150 days after surgery. Within 1–2 years bronchoscopy showed a smooth undiscernible bronchoplasty with a stable open left main bronchus. At 5 years the patient lives an independent life at home with her family.

**Conclusions:**

Surgical treatment of BEF in an extremely complex patient may turn out successfully. It demands careful ethical considerations, comprehensive surgical strategy, multi-disciplinary teamwork, and shared decision making with the patient. The patient presented in this case report is closely followed up with good life quality after 5 years.

**Supplementary Information:**

The online version contains supplementary material available at 10.1186/s13019-024-03287-5.

## Background

Broncho-esophageal fistula (BEF) after radiation therapy for broncho-pulmonary malignancy has previously been described [1]. For most BEF cases, palliative stent treatment is the primary option due to the patient’s underlying conditions. Stents may temporarily improve clinical conditions, however migration of silicone stents and erosions and tissue necrosis from self-expanding stents must be expected. Surgical repair of BEF is the only established definite treatment and has previously been described [1–3], but to the best of our knowledge only in two-lung patients. The risk with surgical treatment of BEF in a single-lung patient is significantly higher than in two-lung patients, and even more after previous chest radiation therapy. Since the prognosis without surgery is poor, surgical treatment should be considered, especially in young patients, even if high-risk. In this report we present a unique case of a young single-lung patient with BEF treated with extensive surgery.

## Case presentation

### Patient history

Eight years after treatment for lung cancer a 40-year-old female developed BEF. Initially she had non-small cell lung cancer stage IIIA and was curatively treated with a right-sided pneumonectomy, lymph node dissection, chemotherapy (3 cycles of cisplatin and vinorelbine) and concomitant radiation (2 Gy × 30) of the mediastinum. Surgery caused a right-sided vocal cord paralysis and the radiation therapy caused esophageal stenoses that were later treated with balloon dilatation. Post cancer treatment, she also developed autoimmune primary adrenal insufficiency (Addison’s disease) and athropic gastritis, which, in addition to preexisting autoimmune hypothyreosis, constitute autoimmune polyendocrine syndrome type 2.

### Mediastinitis, initial interventions and complications

**Fig. 1:**
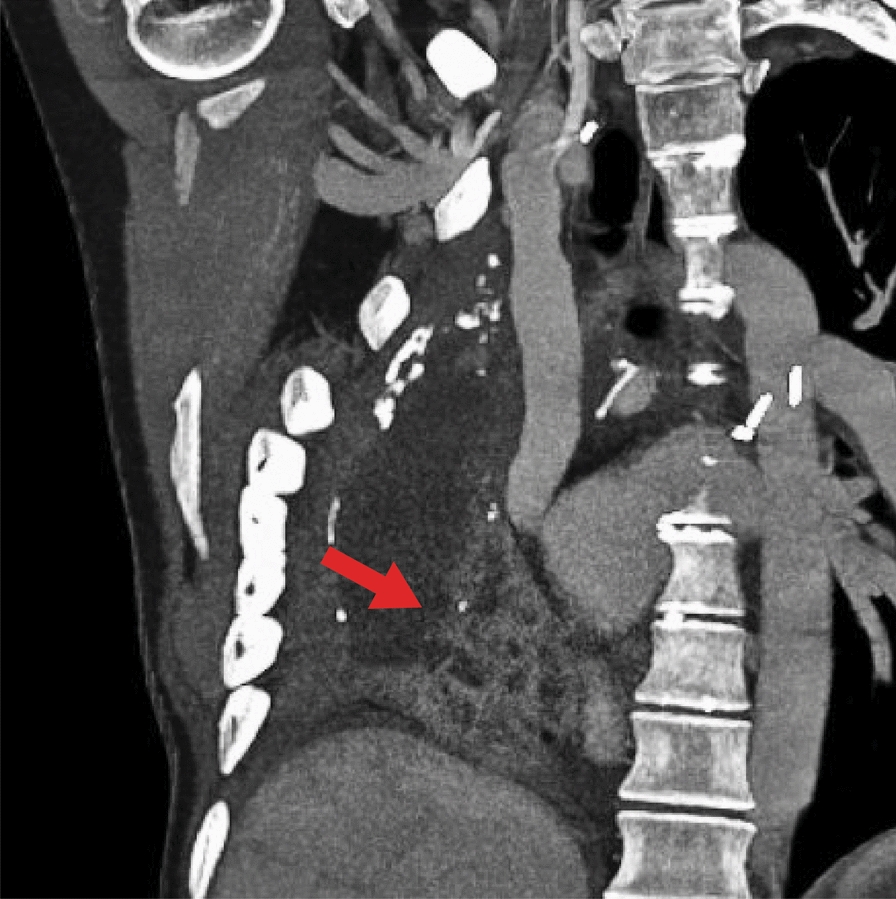
Chest CT scan showing mediastinal abscesses (arrow) between the pneumonectomy cavity, the heart and the diaphragm

Eight years after her cancer diagnosis, mediastinitis manifested due to an assumed esophageal tear (Fig. 1). The infection gradually involved the pneumonectomy cavity following a thoracoscopically created fistula to drain the mediastinum. As infection control was unsatisfactory despite antibiotics and thoracoscopic drainage, a pleuro-cutaneous window and vacuum-assisted treatment followed. *Serratia marcescens* was identified and interpreted as of clinical significance for infection. An acute respiratory collapse required emergency intubation.

### Broncho-esophageal fistula, diagnostics and multi-disciplinary treatment approach

**Fig. 2:**
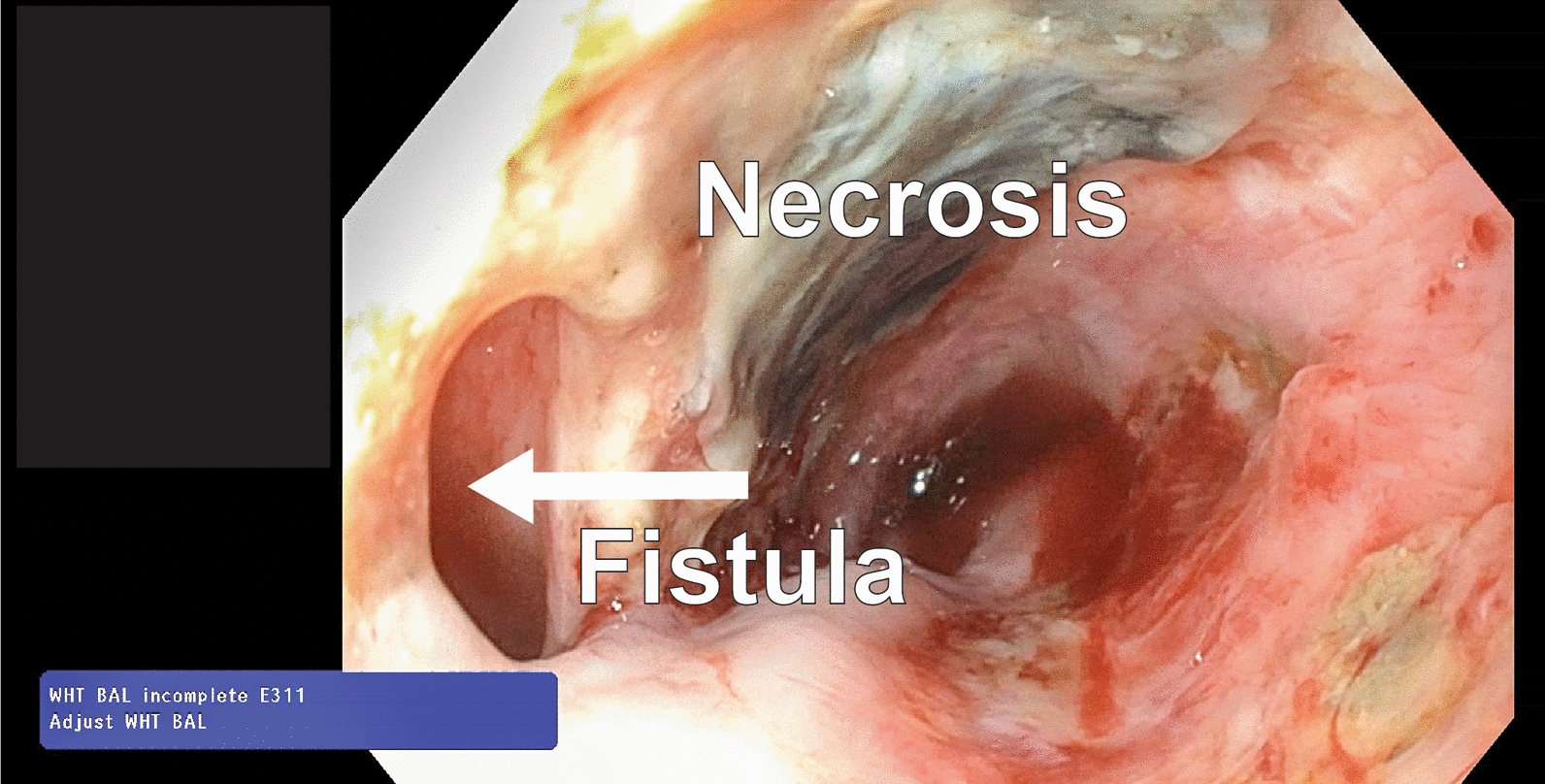
Esophagoscopy detecting necrosis in the anterior wall of esophagus and the broncho-esophageal fistula of 5 mm in diameter (arrow)

**Fig. 3:**
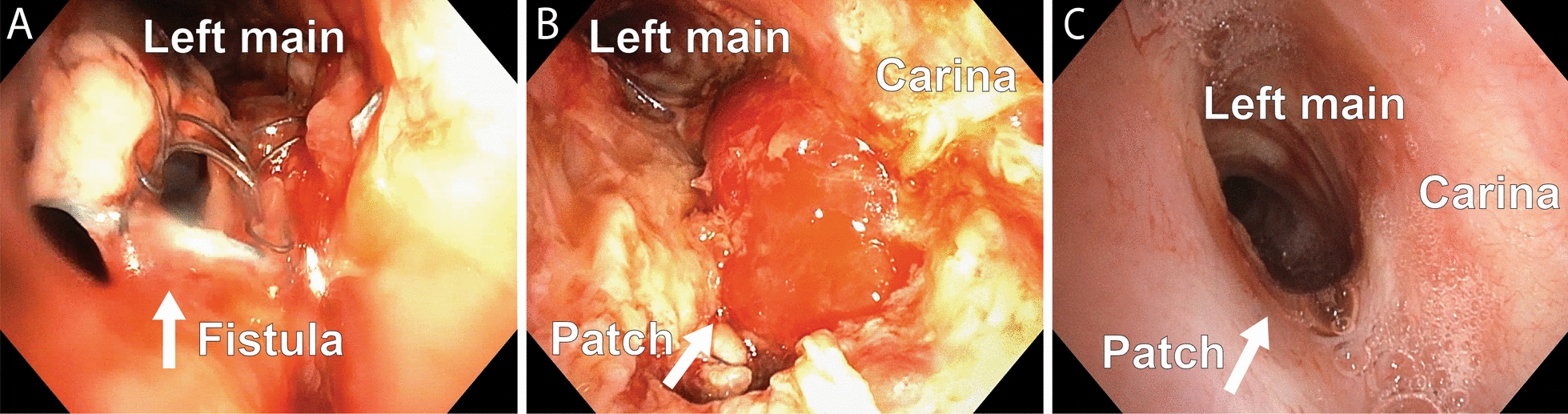
**A**–**C**. Bronchoscopy shows: **A**: The tracheobronchial stent and signs of an accelerating defect (arrow) of bronchus no longer completely covered by the stent, **B**: The left main bronchus and the muscle patch of the bronchoplasty (arrow) after 2.5 weeks, **C**: The bronchoplasty (arrow) completely healed and flush with the original wall after 2.5 years

**Fig. 4:**
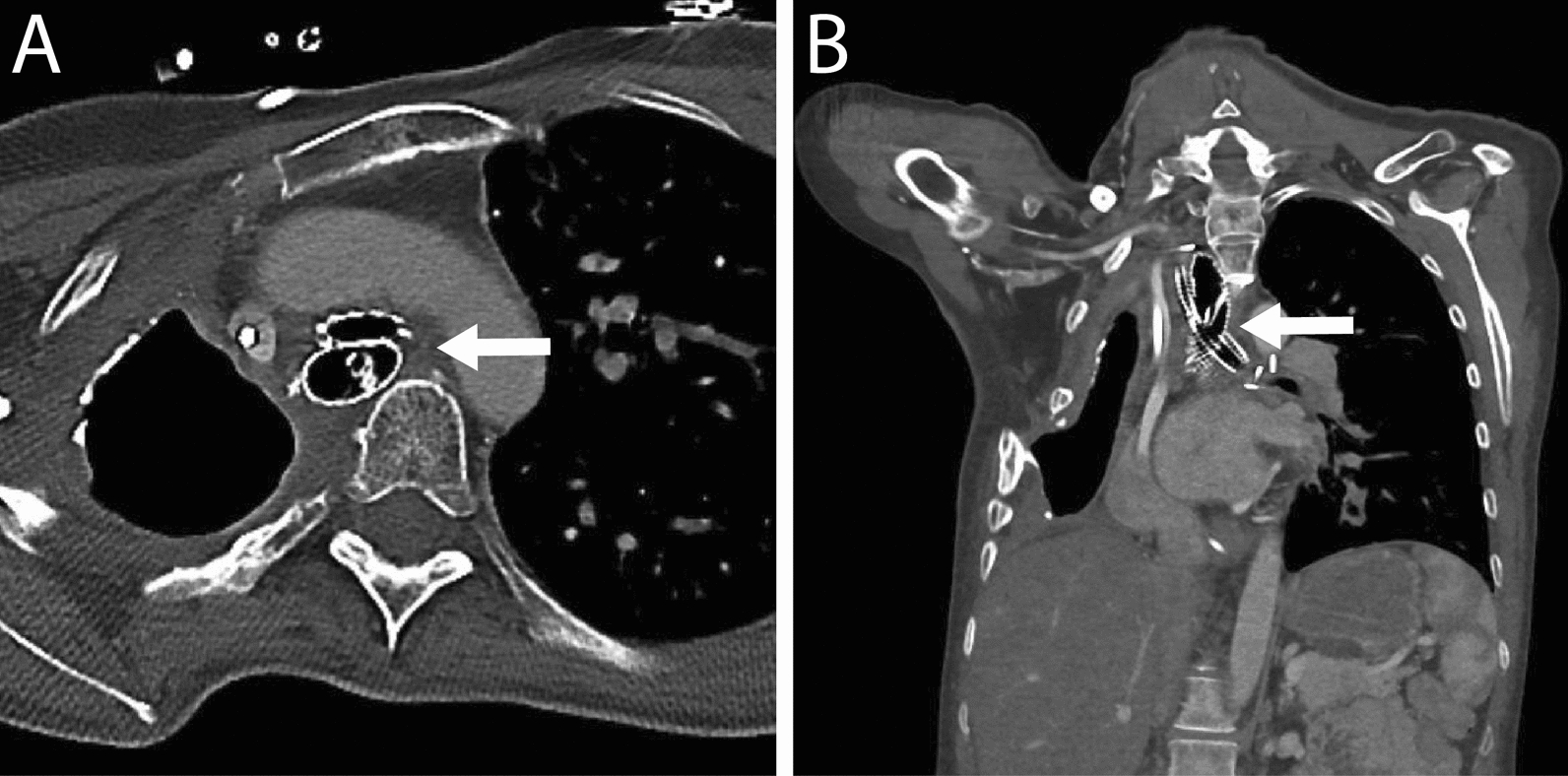
**A**–**B**. Chest CT scan showing locations and crossing of the esophageal and the tracheobronchial self-expanding covered metal stents (arrow)

Broncho- and esophagoscopy revealed a BEF with an initial diameter of 5 mm in the left main bronchus 10 mm distally to the carina, and a necrotic esophagus 24–28 cm below the incisors (Fig. 2). The fistula was sealed by an esophageal stent (covered HANAROSTENT ®, Olympus), a naso-intestinal feeding tube was then placed and the patient was extubated. Due to the critical state, persistent respiratory distress and rare medical condition, a local multi-disciplinary team was established. Tracheobronchial endoscopic examination revealed that the esophageal stent compressed the lumen of the remaining main bronchus. To stabilize the lumen and improve the clinical situation, a tracheobronchial stent (covered Ultraflex® stent, Boston Scientific) was implanted at a collaborative center. Nutrition and mobilization were optimized at the patient ward. However, the stent implants accelerated tissue necrosis of both the esophagus and the membranous part of the bronchus due to compression of the tissue in between the crossing stents (Figs. 3A and 4A, B). A second stent implantation with two overlapping stents did not cover the fistula completely causing increased respiratory distress. See diagnostics in Video 1 (Supplemental).

### Risk assessment and ethical considerations

The treatment options at this point were either palliative treatment with stents or an attempt of definite surgical therapy. Due to the hostile mediastinum, surgical access to the esophagus and bronchus would be difficult, and the risk of a major bleeding from the superior caval vein and the heart during dissection was imminent. Furthermore, to secure oxygenation during bronchial repair, ECMO would be a necessity and contribute to surgical bleeding risk. No similar case was identified in the literature. The patient was discussed with colleagues worldwide. Certain thoracic centers abroad were invited to have the patient referred but declined with reference to the high risk and expected complications.

 Our hospital medical ethics committee was invited to evaluate the treatment considerations and applied a seven-step method for ethical decision-making [4]. The ethics committee agreed that the decision of treatment solution was extraordinary and should be a shared decision between the patient and the multi-disciplinary team. Throughout a comprehensive strategy our multi-disciplinary team considered the chances of successful treatment as realistic despite numerous potential challenges. Noteworthy, the patient and the relatives had throughout the whole course leading up to this been thoroughly informed about our considerations and engaged in the treatment plan. The current situation and treatment options were again formally presented so that a shared decision was assured. A highly motivated patient supported by her family promoted a comprehensive surgical solution at our institution.

### Surgical treatment approach

**Fig. 5:**
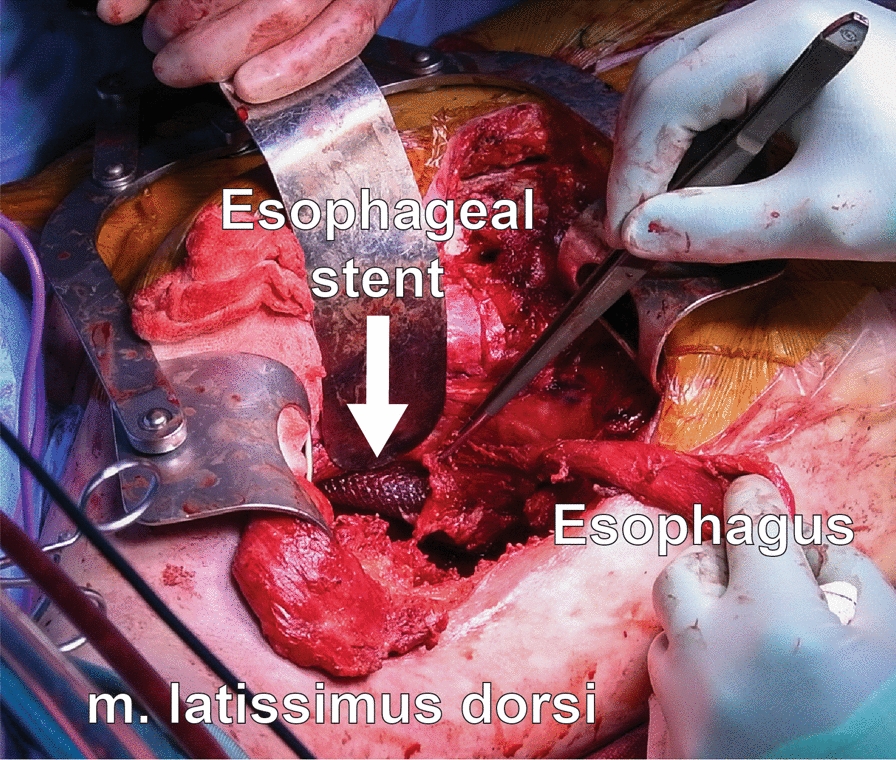
Right-sided thoracotomy, esophagus, esophageal stent and the latissimus dorsi muscle flap

**Fig. 6:**
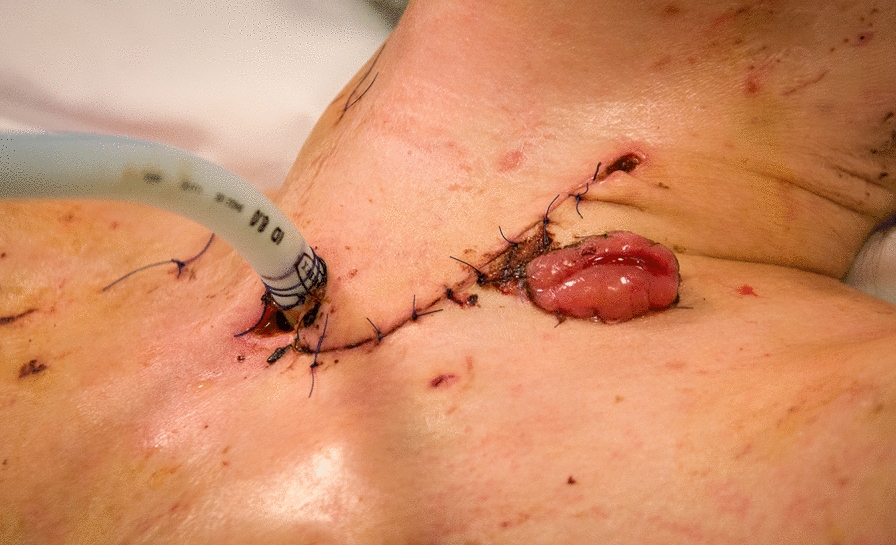
Sutured neck incision access, esophagostoma and customized blue-line tube to cover and protect the bronchoplasty

Surgery was performed 11 months after the onset of the actual mediastinitis and 77 days after the manifestation of BEF (Fig. 5). The surgical approach consisted of four distinct stages: securing oxygenation, extensive thoracoplasty, esophagectomy and bronchoplasty. The intraoperative conditions in the thorax were extremely challenging with displaced anatomy, radiation injury with adhesions and fibrosis in addition to infectious fragile tissue. Each treatment stage was demanding and involved high risk as well as flexibility of the whole team. The surgery was performed throughout three consecutive days with intensive care treatment during the nights. Step by step, the treatment progressed as follows, see Video 1 (Supplemental):

1. A left bronchus intubation and cannulas for femoro-femoral veno-venous ECMO support were established. Femoro-femoral access was chosen due to a stenotic vena cava superior after Hickmann catheterization. Bronchoscopic examinations were repeated for guidance during the procedures.
2. After debridement of infection, most of the chest wall on the right side was removed as part of an extensive thoracoplasty. All available right-sided muscle flaps were prepared. The latissimus dorsi muscle flap was suitable for the bronchoplasty while the intercostal muscle flaps had insufficient elasticity due to previous radiation and infection.
3. Anatomic structures were undiscernible due to adhesions in the mediastinum, so direct access to airways and esophagus was impossible. Endoscopic transillumination of the esophagus was unhelpful, but transhiatal access through a midline abdominal incision facilitated esophageal release all the way to the infiltrate around the left bronchus. A conduit of the larger curvature of the stomach was created for a possible later reconstruction of the GI-tract. A nutritional catheter was introduced to the jejunum. The esophagus was dissected from the left bronchus and trachea by a simultaneous access from the hiatus and the chest. The oral part of the esophagus was skeletonized off the trachea and the upper mediastinum to the thoracic inlet and exteriorized on the left side of the neck as a stoma (Fig. 6).
4. Visualization of the margins of the defect in the left bronchus from the right thoracotomy was highly impaired due to adhesions and fibrotic tissue. To elaborate on the surgical options, a left thoracotomy with intercostal muscle transposition was performed. Due to the mediastinal shift after pneumonectomy and mediastinal adhesions, it did not give additional access to the left bronchus. By combining broncho- and thoracoscopic guidance, in addition to direct visualization into the right thoracic cavity, the defect in the bronchus became sufficiently exposed for repair. The defect now measured 20 × 35 mm and the surrounding tissue was of poor quality. A bronchoplasty was performed by suturing a latissimus dorsi pedicle with 12 interrupted resorbable sutures (Ethicon PDS 4–0), covering the defect. The large thoracoplasty defect was filled with other available muscle flaps.

### Early postoperative clinical course and complications

**Fig. 7:**
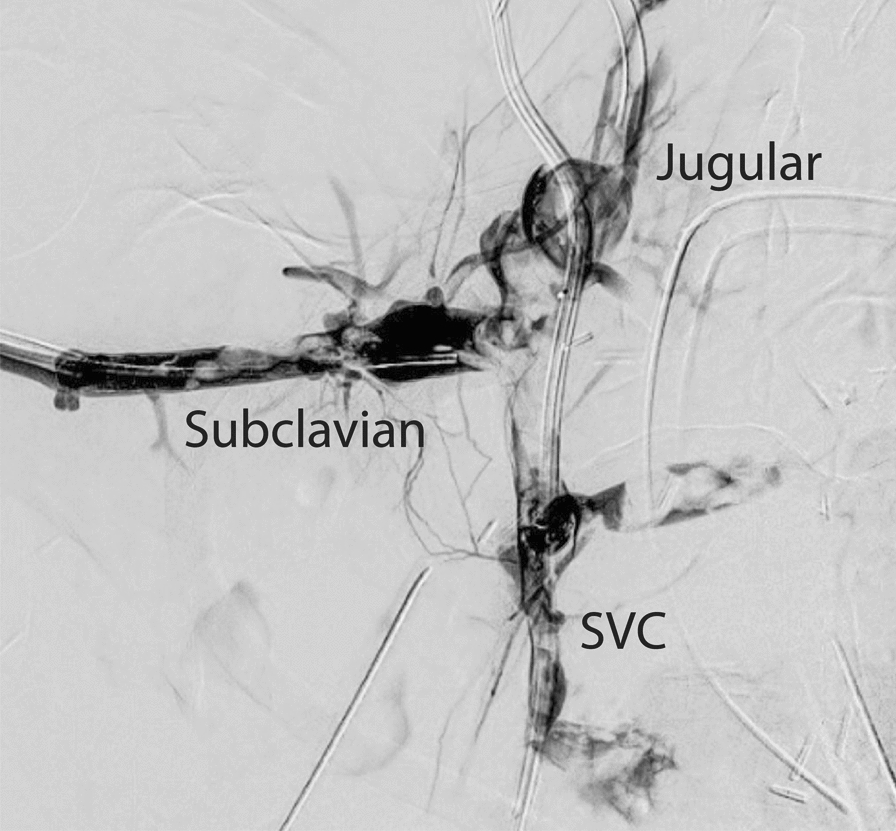
Venography shows the thrombotic occlusion of the upper veins system

Per- and postoperatively, the conditions were complicated by thrombotic occlusion of the vena cava superior, the innominate and the jugular veins (Fig. 7). This caused a massive venous stasis that was relieved by a supplemental ECMO cannula in the jugular vein. Subsequently, the thrombotic event was successfully treated by balloon dilatation through the right brachial vein.

Several reoperations were indicated due to bleeding. Despite this VV-ECMO treatment was continued for 21 days to enable gentle ventilation to relieve the bronchial repair. Furthermore, a customized silicone tracheostomy tube was used to cover and protect the repair area endobronchially. Through a fine catheter parallel to the silicone tube the bronchoplasty wound was irrigated regularly.

Despite the need of a tracheostomy tube for 58 days, making it impossible to talk and suffering from critical-illness polyneuropathy, she eagerly rehabilitated with caring support from the staff. The bronchoplasty was regularly examined and gradually healed (Fig. 3B, C). 150 days after surgery, the patient was discharged to an independent life at home with her family. A tracheostoma was removed after 15 months. In the period following discharge she has had one episode of short-term reintubation due to a viral pneumonia complicated with a nosocomial bacterial superinfection.

### Late outcome

5 years postoperatively the patient has a restrictive but stable lung function impairment (FVC 25%, FEV1 29%, and DLCO 35%), although significantly reduced compared to values before BEF manifestation (FVC 45%, FEV1 40% and DLCO 68%). CT scan has indicated a very slow progressing apical pulmonary fibrosis in the remaining left lung. She uses non-invasive respiratory support at night and has regular right vocal cord injections to optimize glottal closure, respiration and voice quality. The last 4 years the left main bronchus has been wide open with a smooth undiscernible bronchoplasty (Fig. 3C). However, during a planned vocal cord injection 5 years postoperatively, a small sinus (3 mm, not directly related to the bronchoplasty) was observed in the midline of the carina connected to an underlying air loculament. As this observation is asymptomatic, it is currently conservatively treated. The team has discussed performing an esophageal reconstruction for the patient, but this is put on hold to protect her from a possible worsening respiratory function.

## Discussion and conclusions

This report describes a unique case of BEF in a single-lung patient following curative treatment with pneumonectomy and radio-chemotherapy for advanced lung cancer. The patient was 40 years old and had autoimmune comorbidity, a single lung and vocal cord paralysis. Radiation to the thorax and balloon dilatation of the esophagus were interpreted as the main factors for the esophageal tear, mediastinitis and subsequent BEF. Treatment of BEF with self-expanding stents may temporarily improve clinical condition. However, stent erosions and tissue necrosis may complicate the treatment as demonstrated in this case. Silicone stents are known to be prone to migration and were therefore not chosen. Definite treatment is surgical repair (1–3). In our case this involved a very high risk due to a single lung and an extremely hostile thorax. We had established a multi-disciplinary team and prepared for surgery with optimization of infection treatment, nutrition and physiotherapy, as recommended (2). Due to the profound risk of mortality, we also invited the hospital medical ethics committee for an evaluation of the ethical aspects. After fully informed consent and with the patient undergoing veno-venous ECMO support, our team performed esophagectomy, bronchoplasty and extensive thoracoplasty. Prolonged postoperative veno-venous ECMO to secure non-excessive ventilation and a customized tracheostomy tube were strategies that successfully protected the bronchoplasty during initial healing. 5 years follow-up presents an active patient with good life-quality.

To the best of our knowledge, this is the first report of surgical BEF treatment in a single-lung case. The ethical, medical and surgical approach in this case may be instructive and transferable to other patients. It contributes to further knowledge of how to prepare for challenging cardiothoracic treatment and presents applicable surgical techniques and postoperative strategies. In the end, we emphasize that a focused and engaged team as well as a motivated, compliant patient is imperative for success in complex high-risk treatment. The extremely positive fighter-spirit of the patient and her family has been highly impressive and was of utmost importance for the result.

## Supplementary Information

Additional file 1.

Additional file 2.

## Data Availability

Patient data is found in the local electronic patient journal.

## Abbreviations

BEF: Broncho-esophageal fistula
DCLO: Diffusion capacity of the lungs for carbon monoxide
ECMO: Extra-corporeal membrane oxygenation
FEV1: Forced expiratory volume in 1 s
FVC: Forced vital capacity
Gy: Grey

## Declarations

### Guarantor statement

RH is the guarantor of the content of the manuscript and takes responsibility for the integrity of the information and data presented.

## References

[1] Slomowitz E, Tverskov V, Wiesel O. Combined pneumonectomy and esophagectomy for radiation-associated broncho-esophageal fistula. Indian J Thorac Cardiovasc Surg. 2022;38(6):648–50. 35730001 10.1007/s12055-022-01381-8 PMC9192342

[2] Griffo S, Stassano P, Iannelli G, Di Tommaso L, Cicalese M, Monaco M, Ferrante G. Benign bronchoesophageal fistula: report of four cases. J Thorac Cardiovasc Surg. 2007;133(5):1378–9. 17467468 10.1016/j.jtcvs.2006.11.007

[3] Ginesu GC, Feo CF, Cossu ML, Ruiu F, Addis F, Fancellu A, et al. Thoracoscopic treatment of a broncho-esophageal fistula: a case report. Int J Surg Case Rep. 2016;28:74–7. 27689523 10.1016/j.ijscr.2016.09.013 PMC5043393

[4] Miljeteig I, Johansson KA, Sayeed SA, Norheim OF. End-of-life decisions as bedside rationing. An ethical analysis of life support restrictions in an Indian neonatal unit. J Med Ethics. 2010;36(8):473–8. 20663764 10.1136/jme.2010.035535

